# Socio-cultural contribution to medicinal plants assessment and sustainable development: case of antidiabetic and antihypertensive plants in Cameroon

**DOI:** 10.15761/GDT.1000112

**Published:** 2017-01-03

**Authors:** N Tsabang, LWD Tsambang, CG Yedjou, Paul B. Tchounwou

**Affiliations:** 1 Center for Research on Medicinal Plants and Traditional Medicine, Institute of Medical Research and Medicinal Plants Studies. Ministry of Scientific Research and Innovation, Cameroon; 2 Higher Institute of Medical Technoloy, Nkolonodom, Yaounde, Cameroon; 3 Cellomics and Toxicogenomics Research Laboratory, NIH-RCMI Center for Environmental Health, Jackson State University, Jackson, USA; 4 Molecular Toxicology Research Laboratory, NIH-Center for Environmental Health, College of Science, Engineering and Technology, Jackson State University, Mississippi, USA

**Keywords:** Antidiabetic and antihypertensive plants, Ethnobotanical prospections, comparisons between socio-cultural groups, Economic-Botany, Cameroon

## Abstract

Diabetes and hypertension rank among human diseases that are very difficult to control. The medicinal material of Cameroon can provide much information on ethnic folklore practices and traditional aspects of therapeutically important natural products. Cameroon has a very rich cultural diversity with different traditional systems of medicine that need more evidence-based studies on both crude extracts and purified phytomolecules. Therefore, an ethnobotanical study was conducted on 58 socio-cultural population groups living in different phytogeographic units of Cameroon in order to collect various medicinal plants or recipes. A two by two comparison of social-cultural groups of the same phytogeographic unit indicated a significant difference in 86.97% of medicinal plants or recipes comparisons’ cases. A total of two hundred and eight recipes were identified, among which 75 were used for diabetes and hypertension treatment, 74 for hypertension alone, and 59 for diabetes alone. Also, two hundred and three plants were identified among which 33 were cultivated and marketed by 25 farming families engaged in integrated agriculture and selling of antidiabetic and antihypertensive plants to enhance their socio-economic status.

## Introduction

**Figure 1:**
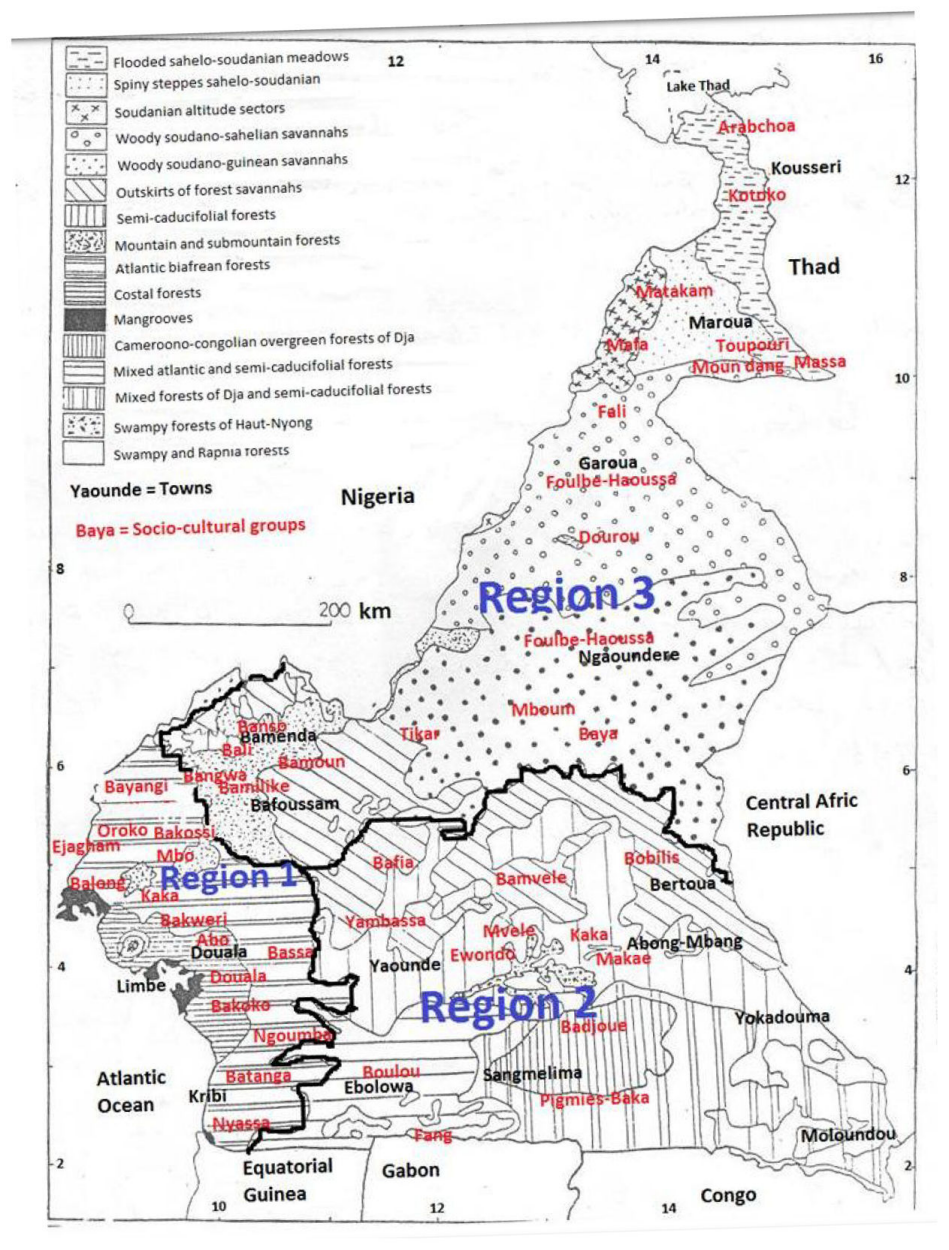
Relationship between socio-cultural groups of interviewees and different phytogeographic units of Cameroon [6,9].

Diabetes and hypertension rank among the chronic diseases that are very difficult to control. In Africa, particularly in Cameroon, traditional healers and a portion of the rural population possess a considerable patrimony of traditional knowledge on medicinal plants, heritage of their ancestral uses. So, great percentage patients in Cameroon live below the poverty line and have many difficulties in meeting fundamental living needs that include health services. As a result, some diabetic and/or hypertensive patients stop treatment and shift to herbal remedies of unconfirmed efficiency and safety [1–3]. Cameroon has a very rich cultural diversity with different traditional systems of medicine that need more evidence-based studies on plants, crude extracts and/or purified phytocompounds. Natural products have been the source of most active ingredients in western medicines. Utilization of natural products as pharmacological tools could lead to the development of a number of new major therapeutically active compounds [4]. Reports on potentially new chemical structures or pharmacological effects of medicinal plant extracts are mushrooming. Many hypoglycemic plants have been identified, and several of them induce the beta cells secretion of insulin. Diabetes and hypertension treatment requires plants with new mechanisms that may be able to reduce insulin resistance, act like injectable insulin at the tissue level, prevent the destruction of beta cells by antibodies or anti-illots beta action, regenerate beta cells, prevent the expression of specific disease-related genes, favor the intestinal absorption of glucose, and correct the abnormalities of the renin-angiotensin II-aldosterone system [1,5]. Therefore, sampling in different tribes may help in the identification of several plants with medicinal properties. The inter communities ethnobotanical information is essential to determine plants diversity, their interspecific variation, and the selection of good useful species. Increasing the awareness of traditional healers to perceive the eventual problems and solutions related to the plant materials used to treat diabetes and hypertension in different tribes, is essential for the identification of the best antidiabetic and antihypertensive plants. In this study, we identified or analyzed the medical potential of indigenous antidiabetic and antihypertensive plant species. We also endeavored to identify numerous medicinal plants by prospecting many traditional systems of medicine in Cameroon [6]. Hence, we answered some key research questions that included: are recipes used against diabetes and/or hypertension varied significantly between socio-cultural groups located in the same phytogeographic unit of the same phytogeoraphic region (Figure 1). In other words, do the uses of medicinal plants easily transmitted from ethnic group to another? Do the populations of socio-cultural groups, living in the same phytogeographic unit use different plants to treat a given disease?.

## Methodology

The process used to collect information on medicinal plants and treatment of diabetes and hypertension began by a field work based on harvest and identification of plants of interest in each ethnic group of interest. During this step ethnopharmacological details on preparation of recipes were collected from traditional healers. This description focused on the mode and the time of preparation, the mode of administration, the posology, the duration of treatment, the undesirable or secondary effects, the toxic effects, the diet and the disease treated. It was followed by the confirmation of botanical identification that was done at the National Herbarium of Cameroon. Voucher specimens were deposited in the Institute of Medical Research and Studies of Medicinal Plants in Yaounde, Cameroon. The research of usage similarity was conducted by comparing 2 by 2 recipes or plants collected in cultural communities living in the same phytogeographic unit. The list of plants and recipes per tribe was constituted. The regrouping of tribes by phytogeographic units was realized by superposing the Letouzey’ vegetation map as described by Achoundong [7] and the location of the different socio-cultural groups of Cameroon [8]. The same methods were used for identifying the different socio-cultural groups. To answer research objective we interviewed a total of 1,131 persons belonging to 58 socio-cultural groups distributed in Cameroon territory (Figure 1).

### Importance economic evaluation of forest plants sold in Yaounde markets

Investigations were carried out on medicinal plants harvested and sold by 25 people in some highly populated market areas of Yaounde. Different plant packagings were previously identified, including bags or strings for all vendors and heaps for retailers (Table 1). This information permits to determine the annual average profits of each category of sellers. Determination of annual average profits of different sellers. There are three categories of sellers: the delivery men, permanent-retailers and collector-retailers. The delivery men, who for most of them were native of the plants’ harvest zones, and relative of permanent-retailers, sold the plant materials collected that include: barks, fruits, whole plants, rhizomes, and leafy stems.

#### Annual average profits of a delivery man

Annual average profits of delivery man = Annual average selling price of all delivery men for all plants **–** their annual transport and hotel fees**/**6 (Number of recorded delivery men)

Annual average profits of a permanent-retailer.

Annual average profits of a permanent-retailer **=** Annual average selling price of all permanent-retailers for all plants **–** their annual average purchase price of all plants**–** [their annual storage fee + their annual guarding fee + their annual tax]**/**14 (Number of recorded permanent-retailers).

Annual average profits of a collector-retailer

Annual average profits of a collector-retailer **=** Annual average selling price of all plants for all collectors-retailers**–** [their annual storage fee + their annual guarding fee + their annual tax +their annual transport fee]**/**5 (Number of recorded collectors-retailers).

## Results and discussion

### Important roles of socio-cultural groups in ethnobotanical assessment

#### Comparisons of recipes between socio-cultural groups located in the same phytogeographic unit

Eighty six point ninety-seven percent (86.97%) of recipes or plants comparisons’ cases between socio-cultural groups located in the same phytogeographic show that tribes use significantly different plants or recipes (Table 2). Individuals of the same origin, the same culture and the same history live by groups more or less close. For this reason a survey conducted in several socio-cultural groups can permit to identify various recipes.

Table 2: Comparisons of antidiabetic and/or antihypertensives plants or recipes used by informants of socio-cultural groups located in the same phytogeographic units in Cameroon.

CF: Coastal forests (Littoral or Atlantic forests with *Lophira alata* and *Saccoglottis gabonensis);* ABF: Atlantic biafrean forest with Cesalpiniaceae; MSMF:Mountain and sub mountain forests; OFS: Outskirts of forests-savannahs; MASCF; Mixed Atlantic and semi-caducifolial forests; MFDSCF: Mixed forests of Dja and semi-caducifolial forests; CCOFD: Cameroon-Congolian Overgreen forests of Dja; SCF: Semi-caducifolial forests; FSSM: Flooded Sahelo-Soudanian meadows; SSSS: Spiny Soudano-Sahelian steppes; SAS: Soudanian altitude sector; WSSS: Woody Soudano-Sahelian savannahs; WSGS: Woody Soudano- Guinean savannhas.

The individuals of the same origin, the same culture and the same history live by groups more or less close. We can conclude that a survey conducted in several socio-cultural groups can allow identifying several plants or recipes. Therefore, floral, climatic, edaphic and socio-cultural differences between regions (2 X 2 compared) are found in the use of antidiabetic and/or antihypertensive plants or recipes. This conclusion is more valuable if the list of recorded plants is representative.

Research on the representativeness of plants recorded for antidiabetic and/or hypertension treatment in Cameroon.

The number of interviewees’ socio-cultural group may be weak, at first sight. But it is in general widely superior to a minimum doorway of four individuals recommended by category for example socio-cultural group, within the context of ethnobotanical surveys [9,10]. The 1.131 interrogated Cameroonians appear negligible when compared with the total population of Cameroon estimated to 13 600 000 of habitants [11]. In this case the rate 0.0832% of participation is very weak. For practical reasons, the minimum doorway of sample difficultly attains 1% of entire population [10]. Many of reticence and distrust from the informants make this minimum doorway more reduced in the area of medicinal plants. That enables us to address the following question: after all, is the list of plants recorded representative of the set of plants used in Cameroon against these two diseases? The answer of this question stands on the study of the growth of the number of recorded plants in relation to the interviewees of each phytogeographic region (Figure 1) Research on the representativeness of plants recorded for antidiabetic and/or hypertension treatment in Cameroon that include Coastal humid forests, Continental humid forests and Guinean and Soudano-zambazian savannahs, in consideration of the fact that the regions compared 2 by 2, show a significant difference. We consider that the interviewees were drawn by lot (without drawback):

14 groups of 20 andone group of 13 in the phytogeographic region 1;
13 groups of 20 andone group of 17 in the phytogeographic region 2;
28 groups of 20 andoneinterviewer in the phytogeographic region 3.

**Figure 2:**
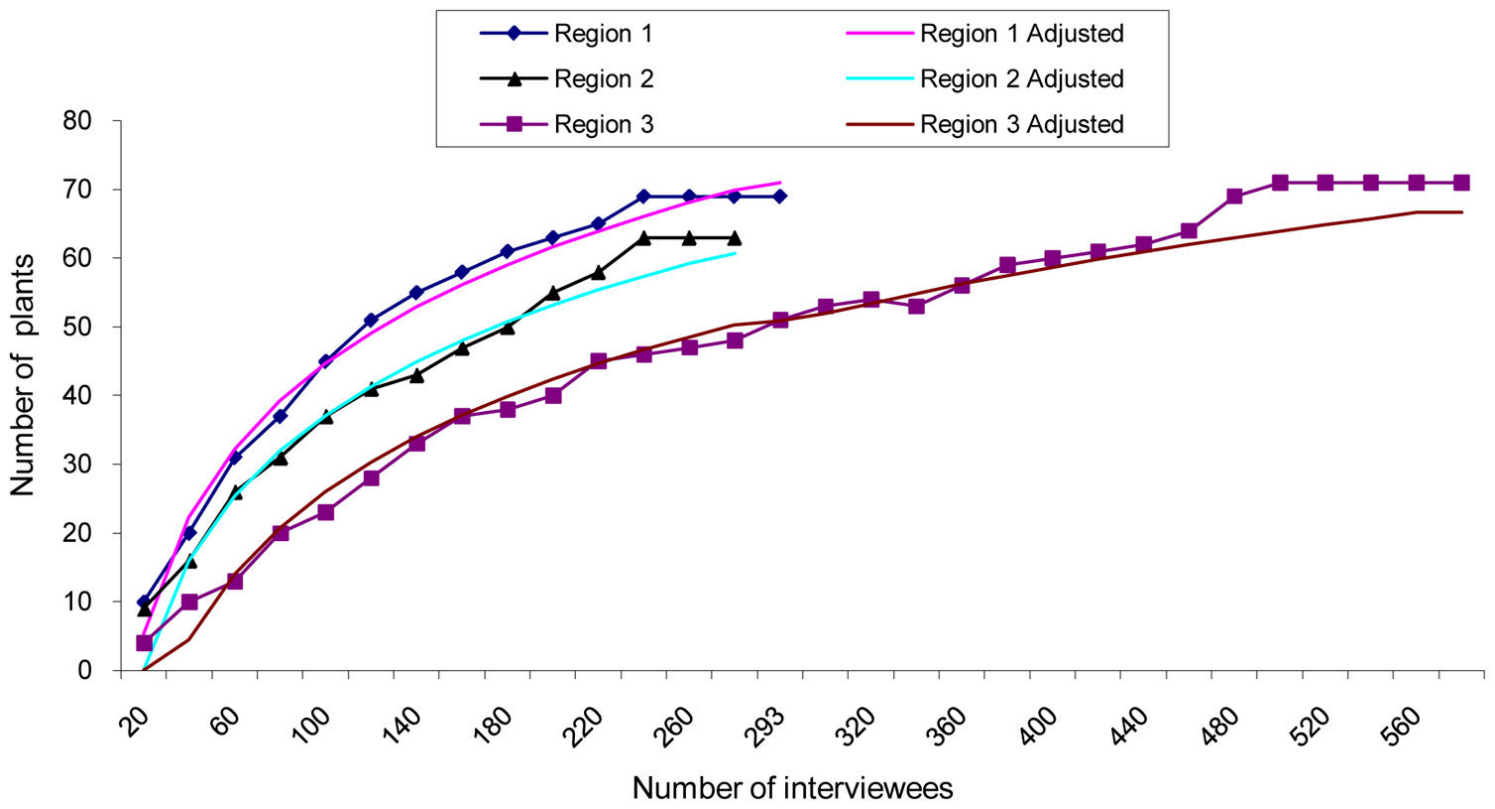
Curves of growth of the number of recorded plants in relation to the number of interviewees by phytogeographic region.

The curbs in each case are illustrated in the Figure 2.

The best adjustment of the trend of each of these curves is a logarithmic function with the following equation:

**Phytogeographic Region 1**: Y = 24.453 ln(X) – 67. 965 with a = - 67. 95; b = 24.453 and the coefficient of correlation R = 0.98;
**Phytogeographic region 2**: Y = 23.103 ln (X) – 69.268 with a = - 69.268; b = 23.103 and the coefficient of correlation R = 0.97;
**Phytogeographic region 3**: Y = 23.584 ln (X) – 82.609 with a = - 82.609 and the coefficient of correlation R = 0.96.

Y is the adjusted number plants and x the number of interviewees, a and b are the adjustment parameters

The analysis of these curves reveals that the number of recorded plants increases with that of the interviewees until a certain level of interviewees (240 – 293) in region 1, (200 – 277) in region 2 and (480 – 561) in region 3. Beyond these levels, any increase of the number of interviewees doesn’t lengthen the list of antidiabetic and/or antihypertensive plants. The list of recorded plants is therefore representative of the set of plants used in Cameroon against the two diseases. We must remark that this is true if the new specimen is distributed in the way that the sample repeated present the same characteristics like the first one, in ethnical point of view; in admitting that each socio-cultural group has a delimited territory, and the same affinity with others that is not taken in to consideration.

Therapeutic, ecological, food, socio-economic importance and statistical significance of recorded plants between the phytogeographic regions

#### Therapeutic importance

By identifying or analyzing the medical potential of indigenous antidiabetic and antihypertensive species, this study has identified numerous medicinal plants by prospecting many traditional systems of medicine in Cameroon. Therefore, two hundred and eight recipes were recorded: Seventy-five (75) are both antidiabetic and antihypertensive, seventy-four (74) are used against hypertension, and fifty-nine (59) are antidiabetic. These recipes were derived from 203 plants belonging to 68 families [9]. Among the 203 recorded plants, 33 have been shown to be very interesting because of their clinical outcomes in 182 patients who used them in self-medication [5].

#### Ecological importance

Wide ecological plasticity plants that include *Momordica charantia, Voacanga africana, Canarium schweinfurthii, Ceiba pentandra, Rauvolfia vomitoria, Allophyllus africanus* and Kigelia africana currently used in most socio-cultural groups.

#### Food, cultivated and local markets sold plants

**F**ifteen recipes are derived from native or introduced plants, which are cultivated for commercial purposes. Native species used also for foods include: *Solamum melongena, Corchorus olitorius, Cucumis metulliferus* and *Vigna unguiculata,* for other medical uses include: *Vernonia glabra*, *Aloe barteri, Aloe buettneri* and *Aloe schweinfurthii.* Introduced plants are *Persea americana, Cymbopogon citratus, Catharanthus roseus, Brassica oleracea, Allium cepa, Allium sativum* and *Citrus grandis*. Wild species which stem barks are sold in the Yaounde markets include: *Mammea africana, Ceiba pentandra*, *Cylicodiscus gabunensis*, *Guibourtia tessmannii*, *Spathodea campanulata*, *Ricinodendron heudelotii, Zanthoxylum zanthoxyloides*, *Entandrophragma* spp, *Newbouldia laevis*, *Morinda lucida*, *Bridelia micrantha*, *Nauclea diderrichii*, *Antrocaryon klaineanum*, *Pterocarpus* spp, *Garcinia lucida*, *Fillaeopsis discophora*, *Hallea stipulosa* and *Pteleopsis hylodendron.* Local markets in urban areas nowadays are refuges for traditional knowledge and places for disseminating of new knowledge and practices about medicinal plants [12]. Like in India, wild edible plants are widely consumed in the daily diet of local people in Cameroon [1]. They are critical for the sustenance of ethnic communities and also as source of income [13,14].

#### Socio-economic importance

Commercialization of antidiabetic and/or antihypertensive plants and possibilities of sustainable development of many tribes.

#### Calculation of storage fees, hotel fees and tax by categories of sellers

Each of the permanent-retailers or collector-retailers pays 200 FCFA for municipal tax per day and 100 FCFA for guarding fee per day, in addition to 40 000 FCFA for annual tax. The year has 360 days for permanent-retailers and 300 days for collector-retailers for which 2 months are used for supplies. The collector-retailers dwell in the collection sites.

The 14 permanent-retailers spend per a year for all the fees: ((200 FCFA + 100 FCFA) x 360 + 40 000 FCFA) x 14 = 2,072,000 FCFA. These expenses are added to 4,918,300 FCFA that is their annual total purchase price of all sold plants. A back is the unit for selling medicinal plants by the delivery man and a heap is the unit of selling for retailers. The value of each unit depends on the importance of the resource and varies from 3,000 FCFA to 10,000 FCFA for a back and from 300 FCFA to 1,000 FCFA for a heap.

The 6 delivery men spend per year 588,895 FCFA for transport and hotel fees. The 5 collectors-retailers spend per year for all the fees: ((200 FCFA + 100 FCFA) x 300 + 40,000 FCFA) x 5 = 650,000 FCFA. They also pay 209.335 FCFA for transport fees. Therefore, their total annual expenses for all plants is 650,000 FCFA + 209,335 FCFA = 859,335 FCFA

#### Table 3

A seller annual average profits on all antidiabetic and/or anti-hypertensive plants sold in Yaounde markets

TAASPP: Total annual average selling price per category of sellers; TAAPPSC: Total annual average purchase price per category of sellers; TAESC: Total annual expenses per category of sellers; TAAPSC: Total annual average profits per category of sellers; AAPSC: Annual average profit of a seller per category.

This Table 3 shows that a collector - retailer earns more than two times than a permanent- retailer and 148,465,500 FCFA more than delivery man. Like the delivery man the collector-retailer doesn’t buy the resources. We realize that the natural harvest and the retail sell are advantageous for the local commercialization of medicinal plants. Therefore, for the same resources sold in the market, personal collection following by retail may be highly recommended. Meanwhile, the formula tax, storage fee and municipal fee significantly decrease the income of permanent-retailers. Other profits are made from the sale of non-antidiabetic and/or anti-hypertensive medicinal plants. In general sellers of medicinal plants can fulfill their financial needs. However, plant harvesting pose a threat to the environment as it becomes more intense in areas far from urban and rural agglomerations.

In Yaounde, prices vary according to season and the distance of the zones of harvest. In Mokolo market the resources are from Lekie Division (Okola, Evodoula and Monatele Subdivisions) and sometimes from Nyong-and Ekele Divisions. In Rond-point Longkak market, plants are from Mbam and Inoubou and Mbam and Kim Divisions. The Mvog-Mbi market is supplying by people of Mefou-Afamba, Mefou-Akono, Ocean and Nyong and So’o Divisions.

#### Comparison of recorded plants in the three phytogeographic regions

The statistical analysis has shown that the list of recorded plants is representative of the set of potential antidiabetic and/or antihypertensive species of Cameroon (Figure 2). Also, a significant difference in plants was observed between the phytogeographic regions compared 2 by 2. Only thirteen plants that includes: *Rauvolfia vomitoria, Momordica charantia*, *Allium cepa*, *Cissus quadrangularis, Laportea ovalifolia; Garcinia afzelii*, *Bidens pilosa*, *Perseaamericana*, *Catharanthus roseus*, *Cymbopogon citratus*, *Margaritaria discoidea*, *Canarium schweinfurthii* and *Voacanga africana* were common for the three regions.

#### Ethnopharmacological preparation and ethnomedical administration of the standard recipes

Almost all the plants cited in this work were prepared and administrated as follow: 100 g of leafy stems of herbal species or 200 g of stem barks were boiled in 4 liters of water, for 25 minutes, each patient was then asked to drink a glass of extract (250 ml) in the morning; mi-day and evening, for 10 days. Specific preparation of each of these plants is detailed in [3,6]. No secondary and undesirable effects were recorded during the treatment. But the prolonged use of *Rauvolfia vomitoria* and *Cissus quadrangularis* can induce vomiting. Maceration of the same quantity of harvested material in the same volume of water, for a whole day, is also allowed for the following plants: *Allium* spp (bulb), *Brassica oleracea* and *Aloe* spp. In these cases, the same posology is respected [9].

## Conclusion

This ethnobotanical study was designed to collect relevant information on medicinal plants or recipes used by various socio-cultural population groups living in different phytogeographic units of Cameroon. From a two by two comparison of social-cultural groups located in the same same phytogeographic unit, it was found that a significant difference exists in 86.97% of medicinal plants or recipes comparisons’ cases. A total of 208 recipes were identified, including 75 that were used for diabetes and hypertension treatment, 74 for hypertension alone, and 59 for diabetes alone. Also, 203 medicinal plants were identified including 33 were cultivated and marketed by 25 farming families who also sold the barks of 18 wild plants. Their engagement in sustainable agriculture and marketing of medicinal plants is helping to improve their socio-economic status. However, many of the antidiabetic and antihypertensive plants are seldom collected and cultivated, although comprehensive assessment has shown that many of them have high clinical outcomes. Hence, further research should be conducted to extract, purify and characterize the active ingredients of these plants. Despite the representativeness of the list of recorded plants identified for diabetes and hypertension treatment in Cameroon, there is a need to improve the methodology for identifying new medicinal plants.

## Figures and Tables

**Table 1:** Summary of socio-economic data questionnaires for a plant.

Number	Country	Date of interview	Day	Month	Year
Identification of informant	Delivery man	Permanent- retailer		Collector- retailer	
**NAME AND SURNAME:**					
Age:					
Sex:					
Village:					
Scientific name of an antidiabetic and/or antihypertensive plant sold in the market		Economic data of an antidiabetic and/or antihypertensive plant sold in the market			
Family:		Average weight of a bag		Average number of heaps per a bag	
		Average purchase price of a kg		Average selling price of a heap	
Genus:		Average purchase price of a bag		Average selling price of a bag	
Species:		Annual average number of bags sold		Annual average number of bags sold	
Conditions of harvest material:		Annual average purchase price of all bags bought by a permanent-retailer = annual average selling price of all bags sold by a delivery man		Annual average resale price of all bags sold by a permanent-retailer	

**Table 2:** Comparisons of antidiabetic and/or antihypertensives plants or recipes used by informants of socio-cultural groups located in the same phytogeographic units in Cameroon. (FSSM: Flooded Sahelo-Sudanian meadows; SSSS: Spiny Sudano-Sahelian steppes; SAS: Sudanian altitude sector; WSSS: Woody Sudano-Sahelian savannahs; WSGS: Woody Sudano-Guinean savannhas)

Phytogeographic regions	Phytographic Units	Number of socio-cultural groups in each phytogeographic units	Number of comparisons’ cases	

			Cases with significant difference observed between plants or recipes	Cases with no significant difference observed between plants or recipes

Region1	CF and Mangroves	6	11	4

	ABF	15	94	11

Region 2	MSMF	8	24	4

	OFS	7	18	3

	FMASC			
	MFDSCF			
	MASCF	6	6	0
	CCODF			

	SCF	4	5	1

Region 3	FSSM	3	3	0

	SSSS	6	11	4

	SAS	2	1	0

	WSSS			
	WSGS:	6	14	1

Total	10	63	187	28

			86.97 %	13.03 %

**Table 3:** Annual average profits gained by sellers on all antidiabetic and/or anti-hypertensive plants sold in Yaounde markets. TAASPP: Total annual average selling price per category of sellers; TAAPPSC: Total annual average purchase price per category of sellers; TAESC: Total annual expenses per category of sellers; TAAPSC: Total annual average profits per category of sellers; AAPSC: Annual average profit of a seller per category

Number of sellers per category	TAASPP	TAAPPSC	TAESC	TAAPSC	AAPSC
14 permanent retailers	12,144,000	4,918, 300	2,072,000	5,153,700	368,121,428
6 delivery men	4,918,300	-	588,895	4,329,405	721,567,500
5 collector-retailers	5,209,500	-	859,335	4,270,165	870,033
